# Mr. Carmichael, on Carbonate of Iron in Cancer

**Published:** 1807-04

**Authors:** R. Carmichael

**Affiliations:** Dublin


*To the Editors of the Medical and Physical Journal.*

Gentlemen,

I Hope the enclosed Letter, which I received from Dr. Barlow, in answer to one I troubled him with, will arrive in time for insertion in your next number, as a note to the paper which I have already transmitted; but should it arrive too late to accompany that communication, I request you will have the goodness to insert it in your publication for April.

I am, &c.

*Dublin, Feb.* 12, 180 R. CARMICHAEL.

---

Sir,

I received your letter, which I was prevented from answering sooner, by as severe a first attack of Gout as ever an unearning mortal had.

As you desired, I enquired of Mrs. R. how long she had the hardness in her breast, before she applied to me. She told me, "she perceived the hard lump in her breast, still increasing in size, for several months; it then became painful, with frequent darting pains, which grew more troublesome every day, for some few months."

When I first saw it, there was a hard irregular tumour, a very small ulcer just at the nipple, and a dark redness of the skin, almost the whole extent of the tumour, which left me in little doubt of all that part becoming ulcerated in a very short time; and from every appearance, with the shooting pains and burning heat which she complained of, I had no doubt in my mind of its being a *confirmed cancer*.

She

She began to take the carbonate of iron, as you directed in your Essay, five grains every fourth or fifth hour; and the ulcer was not only sprinkled with it, but all the discoloured part was covered with it, wet in form of a little poultice.

This was renewed twice a-day. In three or four days, the diseased skin began to separate all round from the sound, which separation still continued to get deeper every day; so that part of the hard tumour was evidently separated to a considerable depth from the sound part of the breast; and from the good matter produced, and the healthy appearance of the surrounding edges, I had every expectation that the tumour would in time, by the application of the carbonate, be entirely thrown off by the sound parts. She went then under your care, and since her return home has continued in good health, with her breast (which she shewed me) perfectly healed, and free from either pain or hardness.

I hope I have been as particular as you wish; if not, any information in my power shall be given with pleasure, by Sir,

Your much obliged very humble Servant,

*Moate, Feb.* 1, 1807. J. Barlow.

---

I cannot pass over in silence my gratitude to this Gentleman, in adopting my remedy, at a time that it was without countenance, and in the readiness with which he makes the above communication. I am under great obligations to many others of our profession; and if I have met with illiberality from a few, who decry my remedy without injury to me, and persist in the use of arsenic without benefit to their patients, I would rather ascribe it to a natural, but often unwarrantable aversion to innovation, than to either petulance or malignity; however, it would be injustice to myself, to pass over an attempt in a late publication, to deprive me of the merits of my discovery, and, perhaps in time, arrogate it to its Editor, or at least, bestow it on Mr. Justamond, who is no longer an object of envy.

In the Edinburgh Medical and Physical Dictionary, under the head of Cancer, there is the following remark. "With regard to the use of iron, it may truly be said, that it has a very considerable effect in supporting the system, and lessening debility, and it will even produce very flattering appearances in some cancerous sores, *more especially those of the face.* It is necessary, perhaps, at the same same time, to employ the spirituous preparation of the same metal as a topic, and in many cases it has been attended with the evident effect of retarding the extension of the cancerous ulcer."

It is true, the Editors of the above work give a long quotation from Mr. Justamond, but they would never have ransacked that author for the above information, if it had not been for the clue afforded them in my Essay, although they think the obligation too trifling to acknowledge. It could have been only from the successful cases related in my work, they learned that this remedy produces "flattering appearances in cancerous ulcers of the face," for Mr. Justamond in every such case which he details, employed *arsenic alone*. And it is extraordinary, that altho' his first motive for employing the remedy, recommended in the German Ephemerides, "was the muriated ammonia used in it, and that he was afterwards convinced that the addition of the steel had a very considerable share in its virtues;" yet with this conviction, Mr. Justamond, in his most favourable case, ascribes the share which iron had in the improvement of his patient, to its invigorating effects on the constitution, and actually applied arsenic to the sore, as the proper remedy for cancer, which he elsewhere terms, "the true antidote against this disease," and uses it in every cancerous ulcer, wheresoever situated. If these Editors be honest in their statement, I have nothing to complain of; they were authorised to omit every name, if they were satisfied in conscience: I was but a plagiary in this discovery, but I should have been indebted to their candour, if they had boldly taxed me with presumption, and given me an opportunity of vindicating my claim. In place of which, they silently seized the hints I gave them, as to the previous use of something like my remedy, and although no fraud might be intended, yet by having dated their title page in 1805, in a few years, it would scarcely be known that the *periodical number*, which contains the disquisition on cancer, did not appear till after my publication in 1806; and on a comparison of dates, little perhaps of the merit of my discovery would have rested with me.

P. S. I would trouble you to correct the following errata in my former paper. In p. 202, line 24, for "but particularly the oxy-phosphate," insert "but that the oxy-phosphate has this property in a far greater degree than the others;" and in p. 209, line 11, for "my discovery," insert "my remedy."

